# Feasibility and utility of transcutaneous spinal cord stimulation combined with walking-based therapy for people with motor incomplete spinal cord injury

**DOI:** 10.1038/s41394-020-00359-1

**Published:** 2020-11-25

**Authors:** Liza V. McHugh, Ashley A. Miller, Kristan A. Leech, Cynthia Salorio, Rebecca H. Martin

**Affiliations:** 1 International Center for Spinal Cord Injury, Hugo W. Moser Research Institute at Kennedy Krieger Institute, Baltimore, MD 21205 USA; 2 Department of Neuroscience, The Johns Hopkins University School of Medicine, Baltimore, MD 21205 USA; 3 Division of Biokinesiology and Physical Therapy, University of Southern California, Los Angeles, CA 90007 USA; 4 Department of Physical Medicine and Rehabilitation, The Johns Hopkins University School of Medicine, Baltimore, MD 21205 USA

**Keywords:** Rehabilitation, Spinal cord diseases

## Abstract

**Study design:**

Prospective case series.

**Objectives:**

To evaluate the feasibility and preliminary efficacy of combining transcutaneous spinal cord stimulation (TSCS) with walking-based physical therapy.

**Setting:**

Hospital-based outpatient center in Maryland, United States.

**Methods:**

Ten individuals with chronic (>1 year) motor incomplete spinal cord injury (iSCI) completed 23 sessions of 2-h therapy over 8 weeks. TSCS was delivered for the first 30 min of each session using a clinically available device with adjustable current. To assess feasibility of the intervention, we tracked pain, adverse events, and participant retention. Preliminary efficacy was assessed by evaluating changes in walking speed, endurance, and quality following the intervention with select functional outcome measures (10-m walk test (10MWT), 6-min walk test (6MWT), timed up and go, and walking index for spinal cord injury II).

**Results:**

We found that the combined intervention was feasible in an outpatient clinical setting. Participants tolerated the TSCS well, with no reports of significant adverse events or other issues (e.g., skin irritation or pain that disrupted training). None of the participants elected to discontinue the study. Participants also showed significant improvements in each measure of walking function following the intervention. Changes in walking speed, as measured by the 10MWT (0.56 ± 0.29 m/s to 0.72 ± 0.36 m/s), exceeded the minimal clinically important difference for individuals with iSCI. Changes in walking quality and endurance, as measured by the 6MWT (149.88 ± 99.87 m to 194.53 ± 106.56 m), exceeded the minimal detectable change for individuals with iSCI.

**Conclusions:**

These results indicate that TSCS is clinically feasible and may be useful as an adjunct to walking-based therapy for adults with iSCI.

## Introduction

Spinal cord injury (SCI) disrupts the transmission of motor and sensory information through the spinal cord. The majority of injuries are motor incomplete spinal cord injury (iSCI) and result in walking dysfunction [1]. Recovery of walking function is a high priority among individuals iSCI and is a common target of physical therapy [2]. Despite significant advances in the field, recovery of independent walking remains elusive for most patients with iSCI.

Intensive gait training is considered the most effective intervention to improve walking function following iSCI [3, 4]. Studies show that this training results in clinically meaningful improvements in gait speed, endurance, balance, and lower extremity strength [1, 5, 6]. However, even with these improvements, significant deficits in walking function persist.

In an effort to potentiate gains in walking function than can be achieved with gait training, previous work in animal and human models has explored pairing intensive gait training with neuromodulatory interventions [7]. One adjuvant intervention that has gained recent attention is spinal cord electrical stimulation. The most well studied form of spinal electrical stimulation to promote recovery of walking function is surgically implanted lumbosacral epidural stimulation [7–10], which acts by directly stimulating the dorsal nerve roots to increase the excitability of interneuronal networks involved in the control of locomotion [7, 10–14]. The current literature has largely described epidural stimulation in individuals with complete SCI, demonstrating that lumbosacral epidural stimulation alone can facilitate reciprocal, step-like movements, and when used in combination with intensive locomotor training can lead to improved walking abilities [8, 14–17].

While this work is exciting, there are limitations to the feasibility of epidural stimulation. First, the surgical placement of the stimulator is invasive and inherently risky [10, 14, 15]. Second, studies demonstrating efficacy of epidural stimulation are time intensive, describing extensive locomotor and gait training for 20–85 weeks after stimulator implantation [8, 10, 14]. Finally, while all participants showed improvement in voluntary motor control, not all recovered durable walking function [10, 14, 15].

Fortunately, a noninvasive form of spinal electrical stimulation has also been shown to impact spinal excitability in individuals with SCI [18–20]. Transcutaneous spinal cord stimulation (TSCS) is thought to increase the excitability of spinal locomotor circuits through dorsal root afferents [18, 21, 22]. Research suggests that this change in excitability enables the brain to utilize functionally silent descending pathways to produce and enhance voluntary movements of paretic limbs [18–20]. Application of TSCS, both in single sessions and repeated over at least 4 weeks, improves standing postural control, gait kinematics, and spinal motor output, when paired with treadmill gait training [17–20]. This suggests that TSCS may be an effective, noninvasive intervention to augment the effects of gait training in motor iSCI.

Despite literature supporting the benefits of TSCS, the effects of pairing TSCS with walking-based therapy in a clinical setting are rarely investigated and largely unknown. Stimulators used in some previous studies utilize a proprietary waveform, not clinically available. The biphasic rectangular wave of these stimulators includes a carrier frequency of 10 kHz, reported to make the stimulation more comfortable than traditional biphasic waveforms [19, 21]. Clinically available stimulators may elicit the same effects, but to date these have not been studied. The purpose of this study was to assess the feasibility and collect preliminary data on the efficacy of providing TSCS with a clinically available waveform as a combined approach to intensive walking-based therapy in an outpatient setting in individuals with motor iSCI.

## Methods

### Participants

Participants were recruited from the International Center for Spinal Cord Injury at Kennedy Krieger Institute. Inclusion criteria for the study were: (1) 18–65 years old; (2) >1 year post SCI; (3) nonprogressive SCI; (4) neurological level at or above T10; (5) tolerates upright position for >30 min; (6) medically stable (no hospitalizations in last 3 months); (7) able to comply with procedures and follow-up; and (8) are legally able to make their own healthcare decisions. Exclusion criteria for participation in the study were as follows: (1) open wounds at stimulation site; (2) pregnant women; (3) ROM limitations impacting gait training; (4) cardiac pacemaker/defibrillator; (5) active cancer diagnosis; (6) currently receiving TSCS; (7) evidence of uncontrolled autonomic dysreflexia; or (8) non-English speaking. Participants were asked to not make any changes to their medications for the duration of the study.

### Study design

**Fig. 1:**
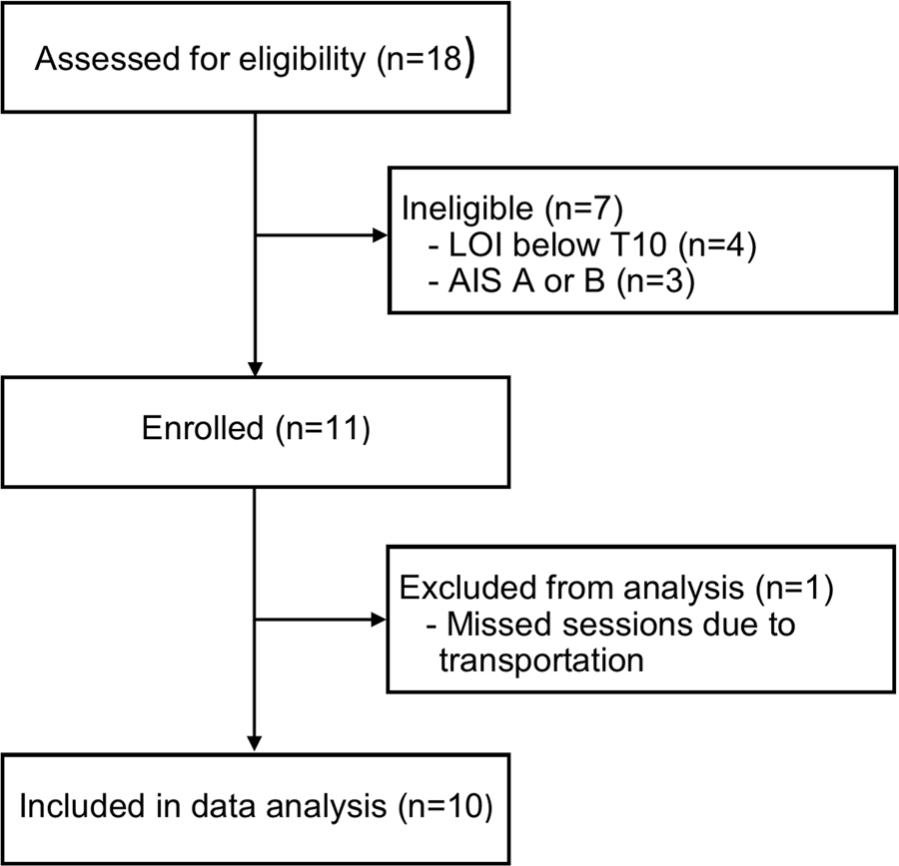
Timeline of training sessions and assessments of walking function.

This prospective case series was a within-participant, repeated measures design (Fig. 1). The intervention consisted of a total of 23 training sessions. Measures of feasibility, pain, skin response, treatment time, and adverse events were collected every session. Measures of walking function were completed at baseline and every sixth session. Post-testing occurred within 48 h of the final training session. All testing was completed without stimulation.

### Walking-based therapy

Therapy sessions were provided for 2 hours three times per week for 8 weeks. TSCS was provided for the first 30 minutes, concurrent with gait activities. Gait activities continued for the remaining 90 minutes without stimulation. This session duration matches other walking interventions with established efficacy, while decreasing the frequency slightly to reduce the burden to the participant, optimize participant retention, and most closely approximate a typical outpatient plan of care [1, 23].

Training sessions were implemented by one of five licensed physical therapists. All therapists followed a general walking-based training protocol with flexibility to address participant-specific gait impairments. The primary focus of each session was walking function.

Intervention activities were divided into four categories: gait-based activities, functional task practice, strengthening exercises, and “other”. Gait-based activities included treadmill walking with body weight support (BWS), over-ground walking with and without BWS, and higher level dynamic gait activities, like tandem walking, step-ups, and marching, walking around obstacles and over uneven surfaces. Functional task practice included the performance of functionally relevant tasks, such as sit to stands from a chair or couch, target stepping, outdoor balance activities, and stair negotiation. Strengthening exercises consisted of a variety of resistance exercises targeting the core and lower extremity musculature. Lastly, “other” accounted for time spent testing, set-up, and participant initiated rest breaks.

### Transcutaneous spinal cord stimulation

At the start of each session, TSCS was applied using a 5 × 10 cm oval electrode placed midline on the skin between spinous processes T11 and T12 and two 7.5 × 13 cm rectangular electrodes placed symmetrically on the skin over the lower abdomen [18, 21]. The Vectra Neo (Chattanooga; Hixson, TN) was used to deliver a symmetrical biphasic rectangular waveform, at 50 Hz and 1 ms, for 30 continuous minutes of stimulation [18]. In previously published work, treatment frequencies of 20–50 Hz were associated with alternating flexion/extension patterns in EMG [16]. In pilot trials, we stimulated at 20, 30, and 50 Hz. At 50 Hz, participants could tolerate greater intensities as compared with 20 or 30 Hz. Though current literature indicates 50 Hz may preferentially target spasticity [24], we did not observe a difference in performance at each frequency. We elected to use 50 Hz to minimize potential discomfort. Intensity was set at the beginning of each session to individual tolerance or submotor threshold, whichever was less. Intensities ranged from 20 to 80 mA and were consistent within sessions but varied between sessions, depending on the participant and their tolerance. Stimulation was adjusted with the patient in sitting, typically at the edge of the mat. Intensity was adjusted to participant’s tolerance or until they reported tingling in the lower extremities. In participants with unreliable sensation, stimulation intensity was increased until oscillating lower extremity movement was observed. Intensity was decreased slightly if the oscillations disrupted the participant’s voluntary motion or efficient gait pattern. Once stimulation was optimized, participants engaged in training as outlined above. Training continued following the stimulation period for the 90-min remainder of the session to take advantage of the priming and lasting neuromodulatory effects provided by the TSCS [19, 21, 24].

### Outcome measures

#### Assessment of feasibility and safety

Pain scores were measured before and after the session using the Numeric Rating Scale for Pain (NRS for Pain) [25]. Pain was rated on a scale of 0–10, with 0 being no pain at all and 10 being the worst pain imaginable [25]. Significant adverse events including, but not limited to, fall incidence, episodes of autonomic dysreflexia, and cardiovascular events were recorded for the duration of the study. Skin response to TSCS was documented at the end of each session to assess skin tolerability. The skin was inspected for signs of mottling or non-blanchable erythema under and around the electrode site to rule out possible tissue injury.

The percentage of possible sessions attended was used as a measure of compliance with the intervention. Therapists’ opinions on ease of administration and comparison to traditional gait-based therapy were also collected.

#### Assessment of effects on walking function

Changes in walking function were assessed with the 10-m walk test (10MWT), 6-min walk test (6MWT), timed up and go (TUG), and walking index for spinal cord injury II (WISCI-II). The 10MWT, 6MWT, and TUG are commonly and widely used functional ambulation outcome measures and have good test–retest, interobserver reliability, and construct validity in ambulatory SCI [26–30]. The WISCI-II, developed specifically for the SCI population, is used to describe walking impairment [27] and provides a more comprehensive consideration of the use of braces and assistive devices not found in the other measures. These measures were chosen to be consistent with a 2008 recommendation of the National Institute on Disability and Rehabilitation Research, to best demonstrate gait improvements in participants with SCI, and the Common Data Elements recommended by the National Institute of Neurological Disease and Stroke and the National Institute of Health [31–33]. For each test, participants were allowed to use a preferred assistive device and/or lower extremity orthoses as needed. Each participant used the same device or bracing at all assessment sessions. All testing was completed without TSCS and participants were not provided any physical assistance during testing. Outcome measures were performed in the same order at each testing point across participants. Participants were offered rest breaks between outcomes as needed.

#### Data analysis

Measures of feasibility are detailed at a group (presented as means ± standard deviations) or individual level, as appropriate. To evaluate changes in walking function following the intervention, we performed separate paired comparisons of pre- and post-intervention assessments for each outcome measure of interest. Paired *t*-tests were used for continuous variables (i.e., 10MWT, 6MWT, and TUG) and Wilcoxon signed-rank was used for ordinal variables (i.e., WISCI-II). For all analyses, *α* level was set to 0.05. Finally, to characterize the time course of changes in walking function, we also present the change scores for each measure at specific time points (visits 6, 12, and 18) during the intervention. These changes are described relative to the established minimal detectable change (MDC) or minimal clinically important difference (MCID) for each measure.

## Results

**Fig. 2:**
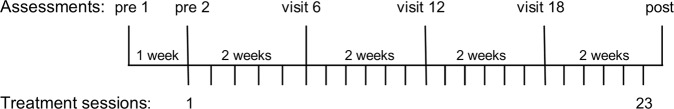
Participant recruitment diagram.

Eighteen people were screened and 11 were enrolled (Fig. 2). We set an a priori threshold to exclude participants from analysis if they missed 10% of the training sessions; one subject was eliminated (missed sessions due to transportation), leaving a final sample of ten. The sample was heterogeneous with respect to neurological level, AIS classification, age, time since injury, and gender. Demographic information is shown in Table 1.

**Table 1:** Demographics.

Subject	Gender	Age (years)	Time since injury (years)	Cause of injury	Neurological LOI	AIS classification
01	M	64	3	Non	T3	D
02	F	22	2	Non	T8	C
03	M	52	11	Trauma	C6	D
04	M	63	57	Trauma	T1	D
05	M	55	18	Non	T4	D
06	F	28	2	Trauma	C4	D
07	F	22	6	Non	C5	C
08	M	40	20	Trauma	C5	D
09	F	60	12	Non	T9	C
10	M	24	3	Non	C7	C

### Feasibility data

Reported pain levels ranged from 0 to 4 on the NRS for Pain with an average of 0.12 ± 0.27 across all participants and sessions, suggesting participants experienced minimal to no pain during the TSCS and associated gait activities. There were no instances in which reports of pain (anything > 0) necessitated termination of the stimulation duration or modification of the interventions.

No significant adverse events, including but not limited to falls, injury, autonomic dysreflexia, or related illness, were reported or observed over the course of the study. Mild, blanchable, erythema under the electrodes was noted on occasion, as is consistent with surface electrical stimulation. These mild skin responses did not limit stimulation duration or impact the physical intervention. No durable or mottled erythema, blisters, or other skin irritation was noted in any participant.

Ten participants completed the 8-week intervention and associated testing. No participant elected to discontinue the study. Participants completed 97.5% of sessions. Only sessions > 90 min were considered complete for the purposes of analysis. Average duration of session across all participants was 112.3 ± 18.7 min of an anticipated 120 min session. Three participants (06, 10, and 11) missed one session for cold symptoms, prearranged vacation, and transportation difficulties. Three participants (02, 07, and 08) stopped sessions early, after 90 min, due to episodes of incontinence. All episodes of incontinence were 60 min or more after completion of the stimulation. Incontinence never occurred during stimulation nor impacted willingness to participate.

Therapists reported that the set-up and administration of the stimulation was easy. They were all familiar with the stimulator from other clinical uses. Therapists reported that the stimulation did not limit or hinder any gait interventions. An additional staff person was required to move the stimulation unit (on wheels) when participants required physical assist for intervention activities.

### Preliminary effectiveness data

**Fig. 3:**
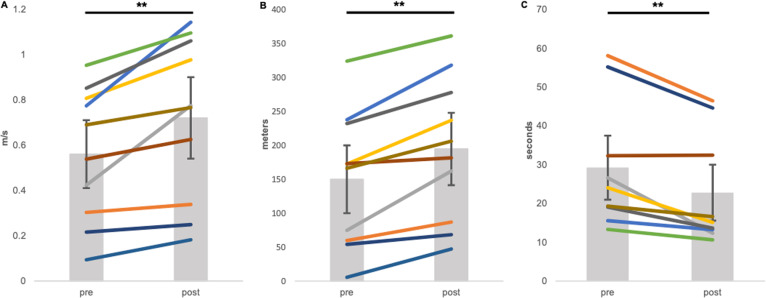
Measures of walking function pre- and post-intervention. Following the interventions, participants demonstrated improvements in walking function demonstrated by significant changes in 10-m walk test (**A**; *n* = 10), 6-min walk test (**B**; *n* = 10), and timed up and go (**C**; *n* = 9). Data are presented as mean ± SD. **Indicates *p* < 0.0001. Lines on each plot represent individual data.

**Fig. 4:**
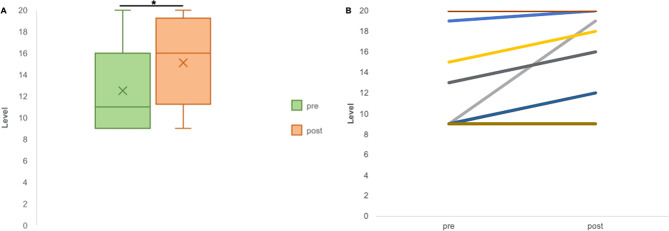
Changes in WISCI-II pre- and post-intervention. Participants demonstrated significant improvements in the walking index for spinal cord injury II (*p = 0.014). Group mean changes (**A**, *n* = 10) and individual changes (**B**, *n* = 10) across the 8 weeks of training are depicted.

Figures 3 and 4 show pre- and post-intervention measures of walking function. Participants exhibited significant improvements in all measures of walking function following the intervention. Specifically, we observed a significant increase in gait speed as measured by the 10MWT, 0.56 ± 0.29 m/s to 0.72 ± .36 m/s, (*t*(9) = −4.08, *p* < 0.0001; Fig. 3A), and endurance as measured by the 6MWT, 149.88 ± 99.87 m to 194.53 ± 106.56 m, (*t*(9) = −5.42, *p* < 0.0001; Fig. 3B). Improvements in functional mobility (i.e., transitional movements, balance, gait speed, etc.) were captured by significant changes in the TUG (*t*(8) = 3.88, *p* < 0.0005; Fig. 3C). One participant (07) was excluded from the TUG analysis, as she was unable to independently complete the required sit to stand transition at baseline, but she showed improvement from session 6 to post-intervention of 53.75 s. Finally, we found a significant group increase on the WISCI-II (*Z*(9) = −2.46, *p* < 0.05; Fig. 4A), suggesting reduction in physical assistance or use of a less restrictive adaptive device or bracing option (i.e., walker to crutches; braces to no braces). Seven out of ten participants improved their individual WISCI-II score by at least 1 point (Fig. 4B), indicating improvement in walking capacity. All participants increased or maintained their score; one participant achieved the maximum score at baseline, precluding improvement on this measure.

### Time course of changes

**Fig. 5:**
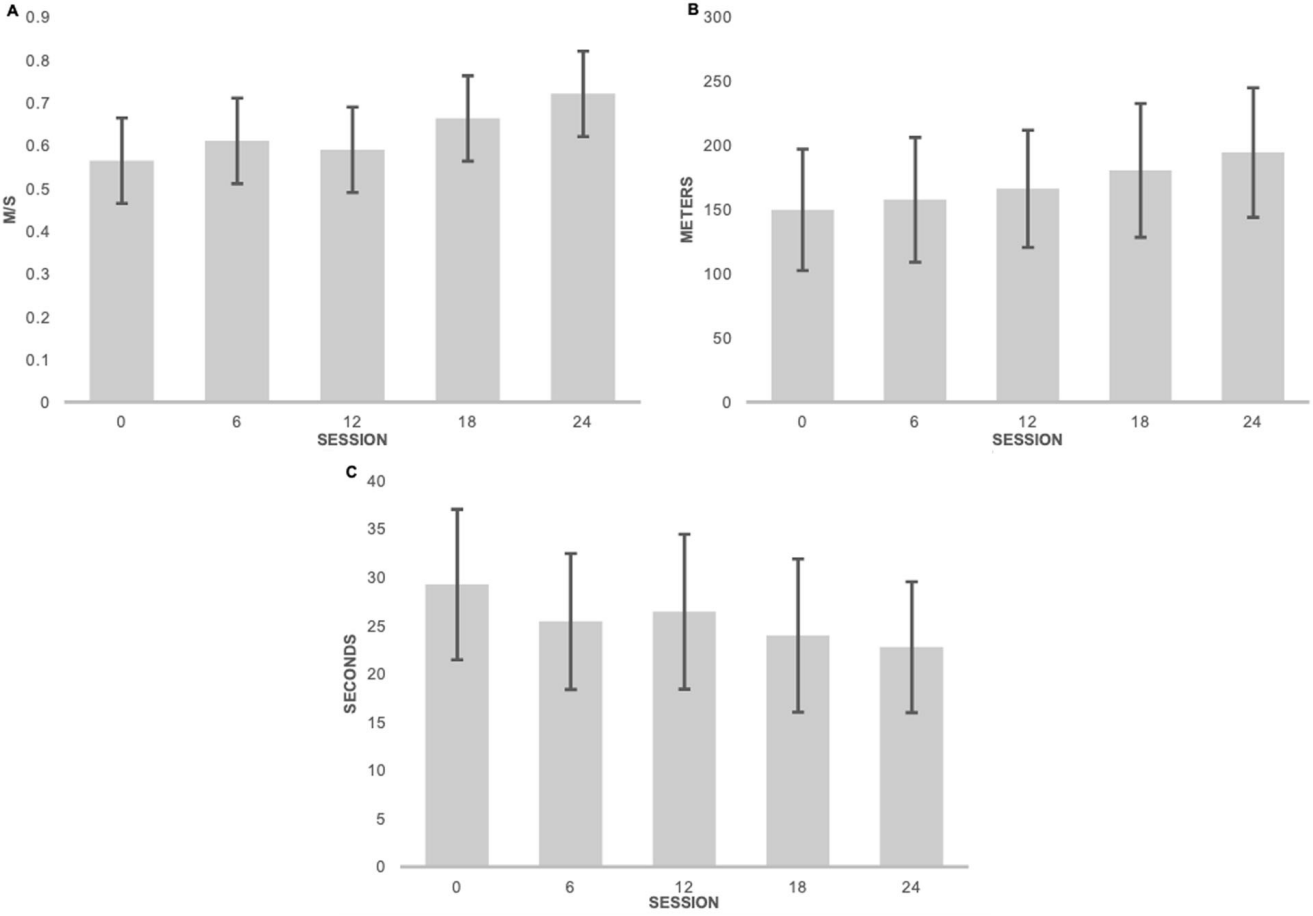
Changes in walking function throughout the intervention. Panels depict the group average (mean ± SD) performance in the 10MWT (**A**, *n* = 10), 6MWT (**B**, *n* = 10), and TUG (**C**, *n* = 9) at 2-week intervals over the course of the intervention. Participants demonstrated change in the 10MWT and 6MWT that exceed the MCID for each measure at or before session 18.

Changes in walking functions at specific time points (sessions 6, 12, 18, and post-intervention) are displayed in Fig. 5. By session 18, group mean changes in gait speed exceeded the MCID reported for the 10MWT in individuals with iSCI (0.06 m/s; Fig. 5A) [34]. This improvement was consistent throughout the group with eight out of ten participants’ individual changes pre- and post-intervention exceeding the MCID. Meaningful changes in endurance occurred earlier in the course of the intervention, with group mean changes in the 6MWT exceeding the MDC for individuals with iSCI by session 12 (54.8 m [26, 35]; Fig. 5B). Changes in walking capacity, as measured by the WISCI-II, also occurred early in the course of the intervention. By session 6, group mean changes exceed the MDC (1 level; [36]) for people with SCI. While this change occurred early, participants continued to improve over the course of the intervention, with a group mean improvement of 2.6 levels on WISCI-II by the post-intervention assessment.

Changes in the TUG across time were more modest. Group mean change, although statistically significant, did not exceed the established MDC for people with SCI (10.8 s; Fig. 5C) [29], suggesting that participants’ walking function improved but their ability to perform transitional movements and specific strength may not have.

## Discussion

In this study, we explored the feasibility of implementing a TSCS protocol in combination with walking-based therapy in individuals with motor iSCI. We provided TSCS through a commercially available electrical stimulation device for the first 30 minutes of each 2-hour session provided three times per week for 8 weeks. Walking-based interventions continued after the stimulation to take advantage of the lasting stimulation-induced changes in spinal excitability [18, 20].

We found that the use of TSCS in combination with walking-based physical therapy is feasible and safe in an outpatient clinical setting. Participants completed 8 weeks of this combined training without report or observation of significant adverse events, despite considerable diversity in clinical characteristic of the sample. The finding that TSCS was feasible and safe in varying degrees of injury enhances its clinical application.

We investigated TSCS with a symmetrical, biphasic waveform via a clinically available device. Previous studies have used an investigational waveform and proprietary device purported to be more tolerable [18, 19, 21]. None of our participants complained of pain nor cited pain as limiting the stimulation intensity or duration, suggesting TSCS may be feasible with a safe, available waveform, enhancing its clinical utility and accessibility for therapists. Electrophysiological studies are still necessary to confirm the neuromodulatory mechanisms of different waveforms currently under investigation.

These results provide preliminary evidence for the effectiveness of this combined training approach to improve walking function in individuals with chronic iSCI. We found that pre- and post-intervention groups mean changes for the 10MWT, WISCI-II, 6MWT met or exceeded the established MDC or MCID for each measure of walking function [26, 34–36]. Many locomotor and activity-based interventional studies for subacute or chronic iSCI report improvement in walking function, specifically related to speed, with similar magnitudes, but often over much longer periods of time [6, 16, 37]. Our participants had been deemed at their maximum functional capacity in traditional therapy, but we demonstrate that that their performance increased at each testing time point, suggesting it is possible for individuals to experience additional real change and progression if training is prolonged. Further research is needed to determine the most effective stimulation waveform, paradigm, and appropriate training dosing and duration, especially in the context of limited healthcare resources.

This was a small, non-powered, non-blinded pilot study and several limitations should be considered when interpreting these data. First, although the changes we observed are significant and most exceed the MCID/MDC for individuals with chronic iSCI, without a control group we cannot attribute these changes to the combined approach versus therapy alone. It is also not clear which factors (age, American Spinal Injury Association Impairment Scale, Neurological Level, baseline walking speed, etc.) predict participants’ improvement in response to the intervention. A larger sample would allow for subgroup analysis, which may elucidate predictive factors. Furthermore, participants engaged in a variety of tasks within a single session, which is typical of clinical therapy sessions. Future studies might limit the walking-based therapy interventions to determine those which are most effective. Finally, as with any intervention in this population, incontinence and transportation are issues. While not related to the intervention, these were issues in our study and could have an impact on the results. Across all sessions, there were three instances of incontinence that occurred more than 30 min after stimulation. Participants did not attribute the episodes of incontinence to the intervention, as incontinence is a common complication of iSCI. Still, we cannot rule this out as an adverse event and it should be more closely investigated in future studies.

These data indicate that TSCS is both clinically feasible and may be a useful adjunct to walking-based therapy for individuals with motor iSCI. Further work is required to adequately assess the benefits of using TSCS combined with physical rehabilitation to improve long-term walking function post iSCI, compared with physical therapy alone.

## Data Availability

The data sets generated and/or analyzed during the current study are available from the corresponding author on reasonable request.
